# The Effects of Tai Chi and Qigong Exercise on Psychological Status in Adolescents: A Systematic Review and Meta-Analysis

**DOI:** 10.3389/fpsyg.2021.746975

**Published:** 2021-11-24

**Authors:** Xuan Liu, Ru Li, Jiabao Cui, Fang Liu, Lee Smith, Xiaorong Chen, Debao Zhang

**Affiliations:** ^1^Faculty of Physical Education, Shenzhen University, Shenzhen, China; ^2^The Cambridge Centre for Sport and Exercise Sciences, Anglia Ruskin University, Cambridge, United Kingdom

**Keywords:** mind-body exercise, psychological well-being, mental, adolescents, review

## Abstract

**Background:** The purpose of this study was to systematically review the effectiveness of Tai Chi and Qigong exercise on adolescents' symptoms of depression and anxiety, and psychological status based on clinical evidences, and to calculate the pooled results using meta-analysis.

**Methods:** A systematic search using seven English and three Chinese databases was initiated to identify randomized controlled trials (RCT) and non-randomized comparison studies (NRS) assessing the effect of Tai Chi and Qigong exercise on psychological status among adolescents. Standardized mean differences (SMD) and their 95% confidence intervals (CI) were used to determine the pooled effect of the intervention. Study quality was evaluated using a Checklist to Evaluate a Report of a Non-pharmacological Trial (CLEAR-NPT) designed for non-pharmacological trials.

**Results:** Four RCTs and six NRS were identified, including 1,244 adolescents. The results suggested a potential beneficial effect of Tai chi and Qigong exercise on reducing anxiety (SMD = 0.386, 95 CI% [0.233, 0.538]) and depression (SMD = 1.937 [95 CI%, 1.392–2.546]) symptoms, and reducing cortisol level (SMD = 0.621 [95 CI%, 0.18–1.062]) in adolescents. Conversely, non-significant effects were found for stress, mood, and self-esteem.

**Conclusions:** The findings of this review suggest Qigong appears to be an effective therapeutic modality to improve psychological well-being in adolescents. Hope future studies will have rigorously designed, well-controlled randomized trials with large sample sizes in order to confirm these findings.

## Background

The *Mental Health* topic launched by the World Health Organization declared that depression is one of the leading causes of disability, affecting ~264 million people worldwide (WHO, 2019). Around one in five children and adolescents in the world suffer from mental health problems, and the onset of nearly half of psychological disorders occurs before the age of 14 (WHO, 2019). Mental health problems in adolescents have been found to contribute to various types of maladaptive behavior, including poor academic performance, violent behavior, teenage pregnancy, drug abuse, self-harm, and even suicide (Das et al., 2016; Dray et al., 2017). Adolescence is a crucial period in which immediate actions should be employed to prevent the impacts of such problem behaviors from persisting into adulthood. Given the public health burden imposed by mental health disorders in adolescents, it is imperious to identify and implement effective interventions.

Currently, many studies have suggested that physical exercise, as an alternative and complimentary therapy, has a positive effect on psychological health (Tsang et al., 2008; Chen et al., 2012; Li et al., 2015; Epps et al., 2019; Garnaes et al., 2019). Qigong exercise is an easily adaptable form of mind-body integrative exercise whose basic components include thoughts concentration, relaxation, meditation, breathing regulation, body posture, and gentle movement (Tsang et al., 2002). Qigong practitioners can experience mood stabilization and mitigation of stress response as Qigong exercise helps decrease physiological arousal and promote relaxation (Wang et al., 2014a). Previous reviews have reported the effectiveness of Tai Chi and Qigong exercise on psycho-physical health among both clinical and non-clinical populations (Cheng, 2015; Webster et al., 2016; Zou et al., 2018; Chang et al., 2019). Additionally, findings of studies supported the beneficial effect of Tai Chi and Qigong exercise on improving physical health (e.g., immune function, cardiovascular health, hypertension) (Qin, 2012; Yu and Chen, 2012; Zhang et al., 2014; Liang, 2018), psychological problems (e.g., anxiety, depression, stress) (Tsai et al., 2003; Lee et al., 2004; Caldwell et al., 2009; Nedeljkovic et al., 2012; Chan et al., 2013; Chang and Wei, 2013), and cognitive function (e.g., executive function) (Tang et al., 2011; Ladawan et al., 2017; Liang, 2018).

Previous systematic reviews examining the effects of Tai Chi and Qigong exercise on psychological status mainly focused on adults and older adults, in particular, with both healthy condition and chronic diseases (e.g., diabetes, depression, cancer) (Lee et al., 2007; Wang et al., 2009; Liu et al., 2015; Sharma and Haider, 2015; Guo et al., 2018; Tong et al., 2018). There has been emerging evidence showing the effectiveness of Tai Chi or Qigong exercise on affecting psychological status in adolescents. For instance, the effect of Tai Chi and Qigong exercise on adolescents' depression, anxiety, stress, and self-esteem have been investigated (Lee et al., 2009, 2013; Terjestam et al., 2010; Sousa et al., 2012; Bao, 2013; Chang et al., 2013; Bao and Jin, 2015; Bao and Niu, 2018; Zhang et al., 2018). However, the findings were not conclusive due to inconsistent findings. Some studies favored the positive effect of Tai Chi and Qigong exercise on reducing stress of adolescents (Terjestam et al., 2010; Zhang et al., 2018), while others yielded no significant results (Lee et al., 2013). Currently, there is a scarcity of systematic reviews and meta-analysis summarizing the effect of Tai Chi and Qigong exercise as intervention on adolescents' psychological status. The purpose of this study was to systematically review the effectiveness of Tai Chi and Qigong exercise on adolescents' psychological status based on clinical evidences, and to calculate the pooled results using meta-analysis.

## Methods

This meta-analysis was conducted in accordance with the Preferred Reporting Items for Systematic Reviews and Meta-Analyses (PRISMA) guideline (Moher et al., 2009).

### Search Strategy

Two reviewers independently searched the literature using the following English and Chinese databases: Medline (*via* PubMed), EMbase (*via* Ovid), PsychINFO (*via* Ovid), Eric (*via* EBSCOhost), SPORTDiscus (*via* EBSCOhost), CINAHL (*via* EBSCOhost), the Cochrane Central Register of Controlled Trials (CENTRAL), the Chinese National Knowledge Infrastructure (CNKI), Wanfang, and the Chinese Scientific Journal (VIP). The searches were conducted from inception through April 2020. The search terms used in this study was based a previous related meta-analysis (Liu et al., 2020): *Qigong, Qi Gong, Ch'i Kung, Qi-gong, Chi Kung, Chi Chung, Qi Chung, Qi-training, Chi Gong, Qigong Massage, Tai Ji, Tai-ji, Tai Chi, Tai Ji Quan, Taiji, Taijiquan, T'ai Chi, Tai Chi Chuan, Tai Chi Chih, Tai Chi Qigong, Baduanjin, Depression, Anxiety, Psychological well-being, Mental, Stress, Mood, Adolescent, Youth, Student, Teenager, Child, Children, Childhood*. Chinese translations of these terms were used in Chinese databases. A complete record of search strings is provided in the Supplementary Material. A manual search of reference lists of all included studies and relevant reviews was conducted to further identify relevant studies.

### Inclusion and Exclusion Criteria

#### Types of Studies

Studies had to be either randomized controlled trials (RCTs) or non-randomized comparison studies (NRS). A study was defined as RCT if the participants were allocated to experimental and control groups randomly; a study was defined as NRS if the allocation of participants was conducted through a systematic sequence without randomization. Studies that did not involve any comparison group or did not report any comparison results between groups were excluded. Meta-analysis, reviews, commentaries, protocols, dissertations, narrative studies, observational or qualitative studies were excluded.

#### Types of Participants

Studies focusing on adolescents with mean age between 12 and 18 years old were included. Given the focus of this review was on general psychological status, rather than on psychopathological symptoms, this review excluded studies on patients diagnosed with major psychiatric disorders (e.g., depression, anxiety, schizophrenia). Studies in patients with chronic illnesses (e.g., asthma, heart disease) were excluded.

#### Types of Intervention

Studies had to use any type of Tai Chi or Qigong as an intervention with comparison such as waitlist control group or other forms of exercise group (e.g., normal P.E classes). Studies integrating Tai Chi or Qigong exercise with other forms of intervention or simply using other forms of intervention such as mindfulness-based training, yoga, and meditation were excluded.

#### Types of Outcome Measures

Studies had to measure the effect of Tai Chi or Qigong on various indicators of psychological status, such as perceived well-being (e.g., self-concept, self-esteem) and psychological distress (e.g., anxiety, depression, stress). Additionally, studies measured physiological indicators, such as cortisol level, which reflect the hormone changes in relation to perceived stress were also included as objective outcomes of psychological status.

### Study Selection and Data Extraction

Two reviewers screened the studies based on the titles, abstracts, and full texts, independently. Discrepancies between the two reviewers (XL, RL) were discussed until consensus was reached. A third reviewer (XC) made the final decision after group discussion if consensus could not be reached. The consistency of abstracts and full-texts screening between the two reviewers was measured using Kappa value proposed by Orwin and Vevea (2009). Two reviewers used a data extraction form to extract relevant characteristics independently, including publication date, study design, location, characteristics of participants (i.e., mean age, gender, sample size), protocol of intervention and comparison group, relevant outcome measures, and main results.

### Quality Assessment

Because of non-pharmaceutical intervention used in included studies, it is difficult to employ a double-blind design. Therefore, a Checklist to Evaluate a Report of a Non-pharmacological Trial (CLEAR-NPT) (Boutron et al., 2005) was used instead of some popular traditional checklist, such as Cochrane Collaboration's assessment tool (Higgins et al., 2008), to evaluate the methodological quality of each non-pharmaceutical study in a more reasonable way. It assessed the quality based on the following criteria: adequacy of randomization, allocation concealment, the availability of intervention details, the appropriateness of care providers' experiences, the adherence of participants, blinding of participants and care providers, blinding of outcome assessors, parallelity of study design, and outcome analysis methods. Since there are difficulties in executing blinding of participants and care providers in non-pharmacological studies, further assessment criteria serve as alternative evaluation regarding the risk of performance bias if there is no blinding or inadequate blinding. A full description of CLEAR-NPT was provided in the Supplementary Material. Some of the included studies did not provide adequate information necessary for the evaluation of each criteria. The reviewers attempted to contact the authors to obtain relevant information. If the information was inaccessible after three attempts of email inquiry, the corresponding criteria was ranked as “unclear.”

### Data Analysis

Comprehensive Meta Analysis (CMA) Version 2 was used to perform the meta-analysis. The *intervention* effect size in each study was presented by the standardized mean differences (SMD) with 95% confidence intervals (CI). Use of SMD allows for the comparisons across included studies where they used different psychometric instruments to measure the same outcome (Deek et al., 2008; Orwin and Vevea, 2009). The included studies were anticipated to be heterogeneous because of the different characteristics of intervention and control types. To account for the potential heterogeneity, a random-effects model was used throughout data synthesis. *I*^2^ statistic was used to assess heterogeneity. Studies with an *I*^2^ statistic of >75% were considered to have a high degree of heterogeneity; studies with an *I*^2^ statistic of 50–75% were considered to have a moderate degree of heterogeneity; and studies with an *I*^2^ statistic of <50% were considered to have a low degree of heterogeneity. Subgroup analysis on primary outcomes based on intervention types (Tai Chi vs. Qigong) was conducted where necessary. Sensitivity analyses were conducted by examining the influence of omitting a single study, respectively, on the overall pooled effect if necessary. It was unnecessary to use funnel plots to assess publication bias because each meta-analysis only included small number of studies.

### Patient and Public Involvement

No patient involved.

## Results

### Search Results

**Figure 1:**
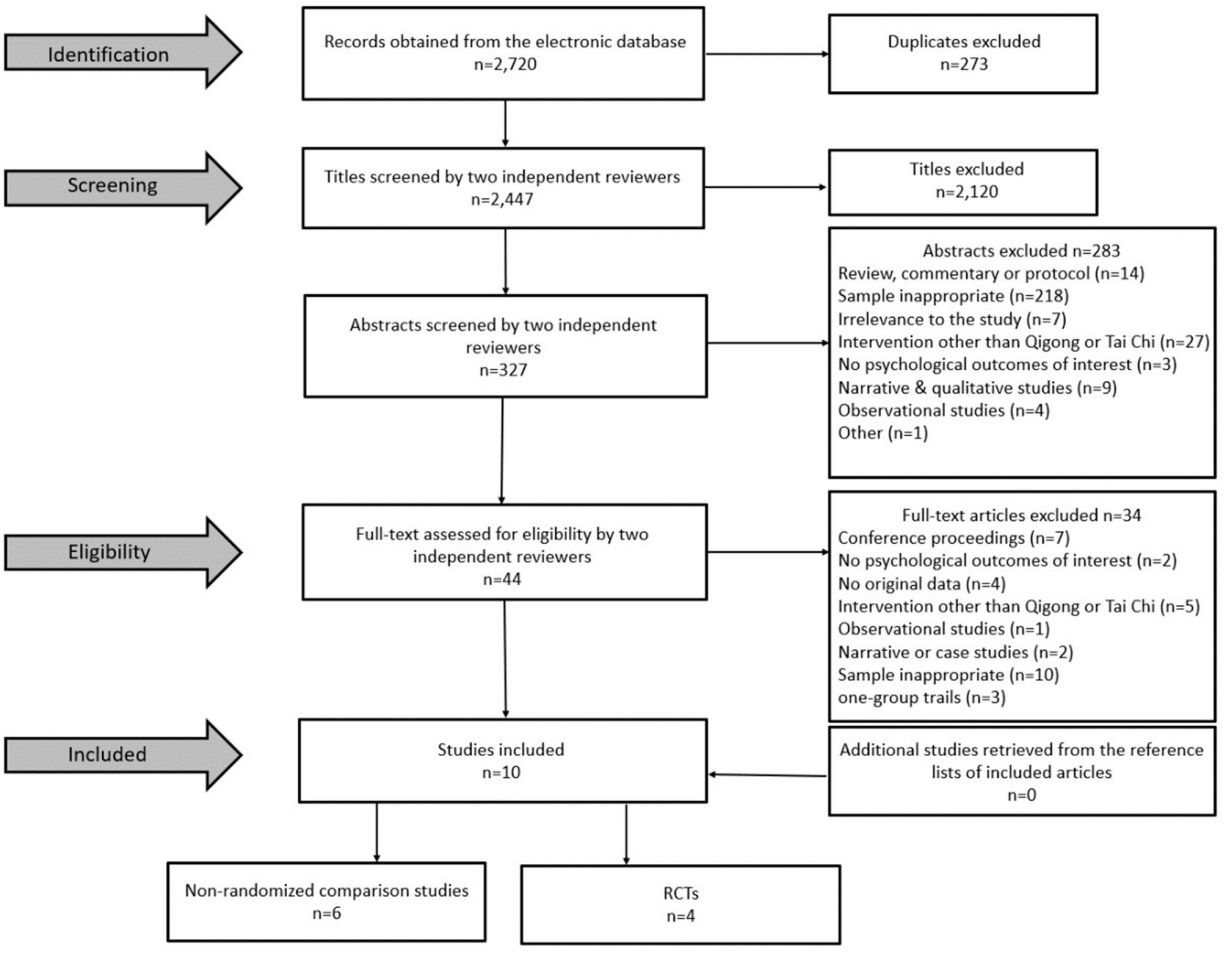
Flowchart of research article selection (RCT, Randomized Controlled Trial).

A total of 2,720 potentially relevant articles were initially screened in the databases. According to inclusion and exclusion criteria, full-texts of 44 articles were assessed for eligibility after removing duplicates, and titles and abstracts screening. Thirty-four studies were further excluded due to the following reasons after full-texts screening: (1) conference proceedings, (2) no psychological outcomes of interest, (3) no original data (4) intervention other than Qigong or Tai Chi, (5) observational studies, (6) narrative or case studies, (7) sample inappropriate, (8) one-group pre-post design. No additional studies were identified from the reference lists of included articles or relevant reviews. The kappa value for screening consistency was 0.93 for abstracts screening, and 0.87 for full-texts screening. Ten studies were finally included in the systematic review and potential meta-analysis (Figure 1).

### Description of Included Studies

Table 1 describes the characteristics of all included studies. The 10 studies were published between year 2009 and year 2018. Three studies identified from Chinese databases were published in Chinese and seven identified from English databases were published in English. The majority (*n* = 7) of the included articles were conducted in mainland of China, Hongkong SAR and Taiwan; the remaining three were conducted in Portugal, Sweden and Korea. Six studies were NRS and four were RCTs. All included studies (*n* = 10) targeted physical healthy adolescents. Instruments used to measure each relevant outcome were also summarized in Table 1.

**Table 1:** Summary of studies included.

**Criteria**		**Number of studies**	**References**
Date of publication	2009–2010	2	Lee et al., 2009; Terjestam et al., 2010
	2011–2018	8	Sousa et al., 2012; Bao, 2013; Chang et al., 2013; Lee et al., 2013; Bao and Jin, 2015; Bao and Niu, 2018; Chen and Zheng, 2018; Zhang et al., 2018
Language of study	Chinese	3	Bao, 2013; Bao and Niu, 2018; Chen and Zheng, 2018
	English	7	Lee et al., 2009, 2013; Terjestam et al., 2010; Sousa et al., 2012; Chang et al., 2013; Bao and Jin, 2015; Zhang et al., 2018
Study location	China (Mainland, Hongkong, Taiwan)	7	Bao, 2013; Chang et al., 2013; Lee et al., 2013; Bao and Jin, 2015; Bao and Niu, 2018; Chen and Zheng, 2018; Zhang et al., 2018
	Others (Portugal, Sweden, Korea)	3	Lee et al., 2009; Terjestam et al., 2010; Sousa et al., 2012
Study design	RCT	4	Lee et al., 2009; Chang et al., 2013; Bao and Jin, 2015; Zhang et al., 2018
	NRS	6	Terjestam et al., 2010; Sousa et al., 2012; Bao, 2013; Lee et al., 2013; Bao and Niu, 2018; Chen and Zheng, 2018
Participants	Physical healthy adolescent	10	Lee et al., 2009, 2013; Terjestam et al., 2010; Sousa et al., 2012; Bao, 2013; Chang et al., 2013; Bao and Jin, 2015; Bao and Niu, 2018; Chen and Zheng, 2018; Zhang et al., 2018
Outcome	Anxiety (PHCSCS, SCL-90-R, MSSMHS, STAI scale)	4	Lee et al., 2009; Bao, 2013; Bao and Jin, 2015; Bao and Niu, 2018
	Depression (PHQ-9, SCL-90-R, MSSMHS)	3	Lee et al., 2009; Bao and Niu, 2018; Zhang et al., 2018
	Stress (PSS-10, General stress test, CPSS)	3	Terjestam et al., 2010; Lee et al., 2013; Zhang et al., 2018
	Salivary cortisol	2	Sousa et al., 2012; Chang et al., 2013
	Mood (FS scale, POMS)	2	Chang et al., 2013; Chen and Zheng, 2018
	Self-esteem (RSE, Self-image test)	2	Terjestam et al., 2010; Chang et al., 2013

*RCT, Randomized Controlled Trial; NRS, Non-randomized comparison studies; PHCSCS, Piers–Harris Children's Self-Concept Scale; SCL-90-R, Self-reported Checklist of 90 items (Physical or Psychological symptom); MSSMHS, Middle School Students' Mental Health Scale; STAI, State Trait Anxiety Inventory; PHQ-9, Nine-item patient health questionnaire depression scale; PSS-10, Perceived Stress Scale 10-item; CPSS, Chinese version of the Perceived Stress Scale; FS, Face Scale; POMS, Profile of Mood States; RSE, Self-Esteem Scale*.

Table 2 shows the characteristics of each included study. Sample sizes ranged from 16 to 312, with a total of 1,244 participants. This included 776 in Tai Chi or Qigong group and 468 in control group. The mean age ranged from 11.75 to 18.4 years old. Several types of Qigong exercise were used, including laughing Qigong, xianggong, baduanjin, and turo qi training. It also used several types of Tai Chi, including Chen-style, mindfulness-based tai chi chuan, and simplified Tai Chi of 24 type. Duration of interventions varied, ranging from 7 weeks to 1 year, with each session lasting for 25–90 min. The frequency ranged from one to seven sessions per week. The control group included waitlist, normal PE classes, China's 8th edition broadcasting gymnastics, placebo group with similar movements.

**Table 2:** Characteristics of included study (*n* = 10).

**References**	**Study design and location**	**Study participants**	**Sample size (pre/post)**	**Intervention (frequency)**	**Control**	**Duration**	**Relevant outcome (measurements)**	**Result**
Lee et al. (2009)	RCT, Republic of Korea	Adolescent (mean age: 13.3 ± 0.1)	EG: 21/21 CG: 27/27	Qigong (Turo Qi training, 40 min/session, twice each week)	Placebo with similar Qigong movement	2 months	(1) Depression & Anxiety (SCL-90-R)	(1) Depression: *p* = 0.06(2) Anxiety: *p* = 0.06
Terjestam et al. (2010)	NRS, Sweden	Adolescent (mean age: 13.15)	EG: 85/53 CG: 71/66	Qigong (25 min/session, twice a week)	Wait list	8 weeks	(1) Well-being at school scale (WBS) (2) Psychologic distress scale (3) Self-image test (4) General stress test	(1) *p* < 0.05(2) *p* < 0.05(3) *p* = 0.078(4) *p* < 0.05
Sousa et al. (2012)	NRS, Portugal	Adolescent (mean age: 11.75 ± 0.55)	EG: 8/8 CG: 8/8	Qigong (every day during 7 weeks, including doing at home on weekend for 30 min from parents)	Wait list	7 weeks	(1) Anxiety Depression and Stress (EADS-C) (2) Salivary Cortisol	(1) *p* = 0.291(2) *p* = 0.606
Bao (2013)	NRS, China	Adolescent	EG1: 103/103 EG2: 92/92 CG: 57/57	EG1: Tai Chi (24-form, 60 min/session, five times per week)EG2: Tai Chi (24-form, 30 min/session, five times per week)	Wait list	1 year	(1) Anxiety (STAI)	(1) EG1: *p* = 0.004 EG2: *p* = 0.003
Chang et al. (2013)	RCT, Taiwan	Adolescent (7th grade students)	EG: 34/34 CG: 33/33	Qigong (60 min/session, once a week)	Wait list	8 weeks	(1) Self-esteem (RSE) (2) Humor (CHS) (3) Mood (FS) (4) Salivary Cortisol	(1) *p* = 0.74 (2) *p* = 0.01 (3) *p* = 0.04 (4) *p* = 0.058
Lee et al. (2013)	NRS, Hongkong, China	Adolescent (mean age: 13.4; 11–16)	EG: 32/32 CG: 37/37	Tai Chi (Chen-style, 80 min/session, once per week)	Wait list	10 weeks	(1) Stress (PSS-10)	(1) *p* = 0.726
Bao and Jin (2015)	RCT, China	Adolescent (mean age: 14.4 ± 0.66)	EG: 80/73 CG: 80/69	Tai Chi (60 min/session, five times per week) including the summer and winter holidays	Broadcasting gymnastics	1 year	(1) Self-concept (PHCSCS)	(1) Self-concept: *p* < 0.001(2) Anxiety: *p* < 0.01
Bao and Jin (2015)	NRS, China	Adolescent (mean age: 12.55 ± 0.729)	EG: 239/239 CG: 73/73	Tai Chi (24-form, 30 min/session, five times per week)	Wait list	1 year	(1) Mental health (MSSMHS)	(1) Depression: *p* = 0.000(2) Anxiety: *p* = 0.000
Chen and Zheng (2018)	NRS, China	Adolescent (middle school students)	EG (Boys): 25/25 EG (Girls): 25/25 CG (Boys): 25/25 CG (Girls): 25/25	Qigong (Baduanjin, 60 min/session, three times per week)	Wait list	4 months	(1) Mood (POMS)	(1) Boys: *p* = NS Girls: *p* = NS
Zhang et al. (2018)	RCT, China	University student (mean age: 18.4 ± 2.01)	EG: 32/32 CG: 32/30	Tai Chi (24-form, 90 min/ session, twice a week)	Normal PE classes	8 weeks	(1) Depression (PHQ-9) (2) Mindful Attention and Awareness (MAAS) (3) Stress (CPSS)	(1) *p* < 0.001(2) *p* < 0.001(3) *p* < 0.001

*RCT, Randomized Controlled Trial; NRS, Non-randomized comparison studies; EG, Experiment Group; CG, Control Group; PE, Physical Education; SCL-90-R, Self-reported Checklist of 90 items (Physical or Psychological symptom); WBS, Well-being at school; EADS-C, Anxiety Scale, Depression and Stress for Children; STAI, State Trait Anxiety Inventory; RSE, Self-Esteem Scale; CHS, Chinese Humor Scale; FS, Face Scale; PSS-10, Perceived Stress Scale 10-item; PHCSCS, Piers–Harris Children's Self-Concept Scale; MSSMHS, Middle School Students' Mental Health Scale; POMS, Profile of Mood States; PHQ-9, Nine-item patient health questionnaire depression scale; MAAS, Mindful Attention and Awareness Scale; CPSS, Chinese version of the Perceived Stress Scale; NS, Not Significant*.

### Study Quality Assessment

Table 3 presents the methodological quality of all included studies. The generation of random allocation was only adequately conducted in one study (Lee et al., 2009). Although four studies were RCTs (Lee et al., 2009; Chang et al., 2013; Bao and Jin, 2015; Zhang et al., 2018), only two of them performed allocation concealment (Lee et al., 2009; Zhang et al., 2018). Five of the included studies reported the details of administration of intervention (Terjestam et al., 2010; Chang et al., 2013; Lee et al., 2013; Bao and Jin, 2015; Zhang et al., 2018). Three studies clarified the care providers' experience or skills (Lee et al., 2009, 2013; Chang et al., 2013). Dropout rate calculation was available in one study (Terjestam et al., 2010). Only one study successfully blinded the outcome assessors and participants (Zhang et al., 2018), and the blindness of care providers was conducted in four studies (Chang et al., 2013; Lee et al., 2013; Bao and Jin, 2015; Zhang et al., 2018). Two studies implemented parallel design between intervention and comparison group (Lee et al., 2013; Zhang et al., 2018). Five studies analyzed main outcomes according to the intention-to-treat principle (Lee et al., 2009, 2013; Sousa et al., 2012; Chang et al., 2013; Chen and Zheng, 2018).

**Table 3:** Critical appraisal of included studies (*n* = 10).

**Criterion**		**Study reference**								
	**Lee et al. (2009)**	**Terjestam et al. (2010)**	**Sousa et al. (2012)**	**Bao (2013)**	**Chang et al. (2013)**	**Lee et al. (2013)**	**Bao and Jin (2015)**	**Bao and Niu (2018)**	**Chen and Zheng (2018)**	**Zhang et al. (2018)**
1. Was the generation of allocation adequate?	Y	N	N	N	N	N	U	N	N	Y
2. Was the treatment allocation concealed?	Y	N	N	N	N	N	U	N	N	U
3. Were details of the intervention administered to each group made available?	U	Y	U	N	Y	Y	Y	N	N	Y
4. Were care providers' experience or skills in each arm appropriate?	U	U	U	U	Y	Y	U	N	N	Y
5. Was participant (i.e., patients) adherence assessed quantitatively?	U	N	U	N	N	Y	U	U	U	U
6. Were participants adequately blinded?	U	N	N	N	N	N	N	N	N	Y
6.1 If Participants were not adequately blinded:										
*6.1.1 Were other treatments and care (i.e. co-interventions) the same in each randomized group?*	U	N/A	N/A	N/A	N/A	N/A	U	N/A	N/A	_
*6.1.2 Were withdrawals and lost-to-follow-up the same in each randomized group?*	Y	N	Y	U	Y	Y	Y	U	U	_
7. Were care providers for the participants adequately blinded?	N	U	N	N	Y	Y	Y	N	N	Y
7.1 If care providers were not adequately blinded:										
*7.1.1 Were other treatments and care (i.e., co-interventions) the same in each randomized group?*	Y	N/A	N/A	N/A	_	_	U	N/A	N/A	_
*7.1.2 Were withdrawals and lost-to-follow-up the same in each randomized group?*	Y	U	Y	U	_	_	Y	U	U	_
8. Were outcome assessors adequately blinded to assess the primary outcomes?	N	U	N/A	U	N	N	N	U	U	Y
*8.1 If outcome assessors were not adequately blinded, were specific methods used to avoid ascertainment bias?*	U	U	_	U	N	N	N	U	U	_
9. Was the follow-up schedule the same in each group? (parallel design)	N	N/A	N/A	N/A	N	Y	N/A	N/A	N	Y
10. Were the main outcomes analyzed according to the intention-to-treat principle?	Y	N	Y	N	Y	Y	N	N/A	Y	N

*N, Not reported; N/A, Not applicable; Y, Yes*.

### Primary Outcomes

#### Anxiety

Four studies examined the effect of Tai Chi or Qigong exercise on anxiety (Lee et al., 2009; Bao, 2013; Bao and Jin, 2015; Bao and Niu, 2018). One study used a three-arm design with two Tai Chi groups of different intensity compared to a waitlist control group (Bao, 2013). These two comparisons were, therefore, conducted separately in meta-analyses. Participants assigned to a Tai Chi or Qigong exercise group perceived less level of anxiety than did those in the control group (SMD = 0.386, 95% CI [0.233, 0.538]). Heterogeneity among studies was high (*I*^2^ = 90.965%).

**Figure 2:**
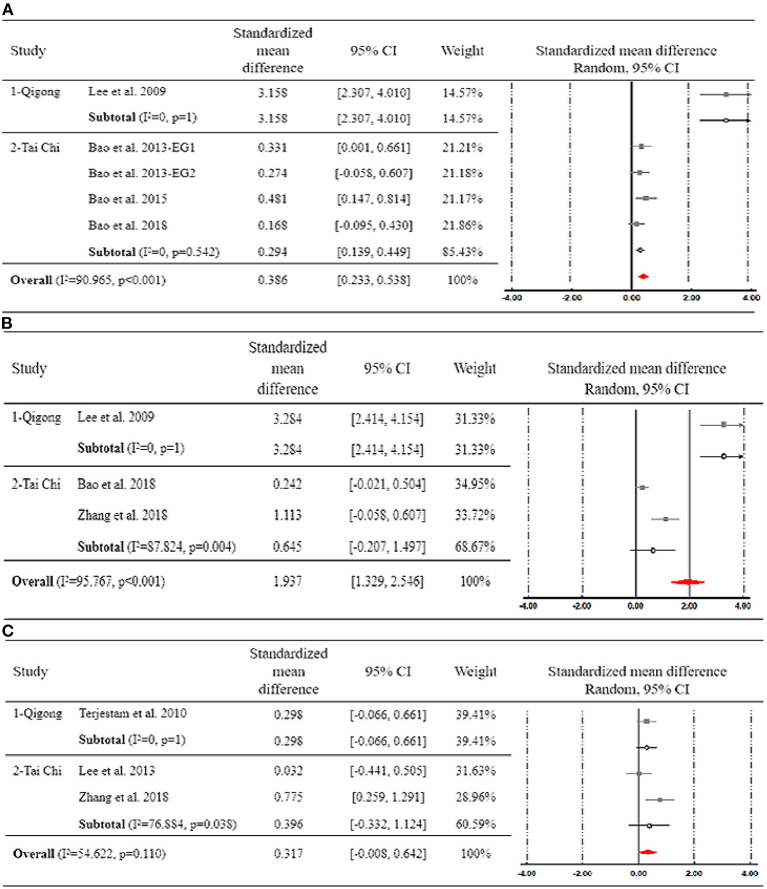
Forest plot for subgroup analysis regarding the effect of Tai Chi and Qigong on **(A)** anxiety, **(B)** depression, and **(C)** stress.

The subgroup analysis based on the intervention type showed that, compared with adolescents who practicing Tai Chi (SMD = 0.294, 95% CI [0.139, 0.449]), those engaging in Qigong exercise perceived greater improvement in anxiety (SMD = 3.158, 95% CI [2.307, 4.010]) (Figure 2A). The difference between groups was statistically significant (*p* < 0.001).

#### Depression

Three studies examined the effect of Tai Chi or Qigong exercise on depressive symptoms (Lee et al., 2009; Bao and Niu, 2018; Zhang et al., 2018). Their pooled results confirmed the significant effect of Tai Chi and Qigong on reducing depression among adolescents (SMD = 1.937, 95 CI% [1.329, 2.546]). Heterogeneity among studies was high (*I*^2^ = 95.767%).

The subgroup analysis based on the intervention type showed that, compared with adolescents who practicing Tai Chi (SMD = 0.645, 95% CI [−0.207, 1.497]), those engaging in Qigong exercise perceived greater improvement in depression (SMD = 3.284, 95% CI [2.414, 4.154]) (Figure 2B). The difference between groups was statistically significant (*p* < 0.001).

#### Stress

Three studies measured self-perceived stress as the primary outcome (Terjestam et al., 2010; Lee et al., 2013; Zhang et al., 2018). Their pooled results showed that Tai Chi and Qigong had an insignificant effect on stress compared to various controls (SMD = 0.317, 95 CI% [−0.008, 0.642]), with substantial degree of heterogeneity (*I*^2^ = 54.622%).

The subgroup analysis based on the intervention type showed that both Tai Chi and Qigong exercise could not significantly reduce stress. The difference between groups was not significant (*p* = 0.110) (Figure 2C).

**Figure 3:**
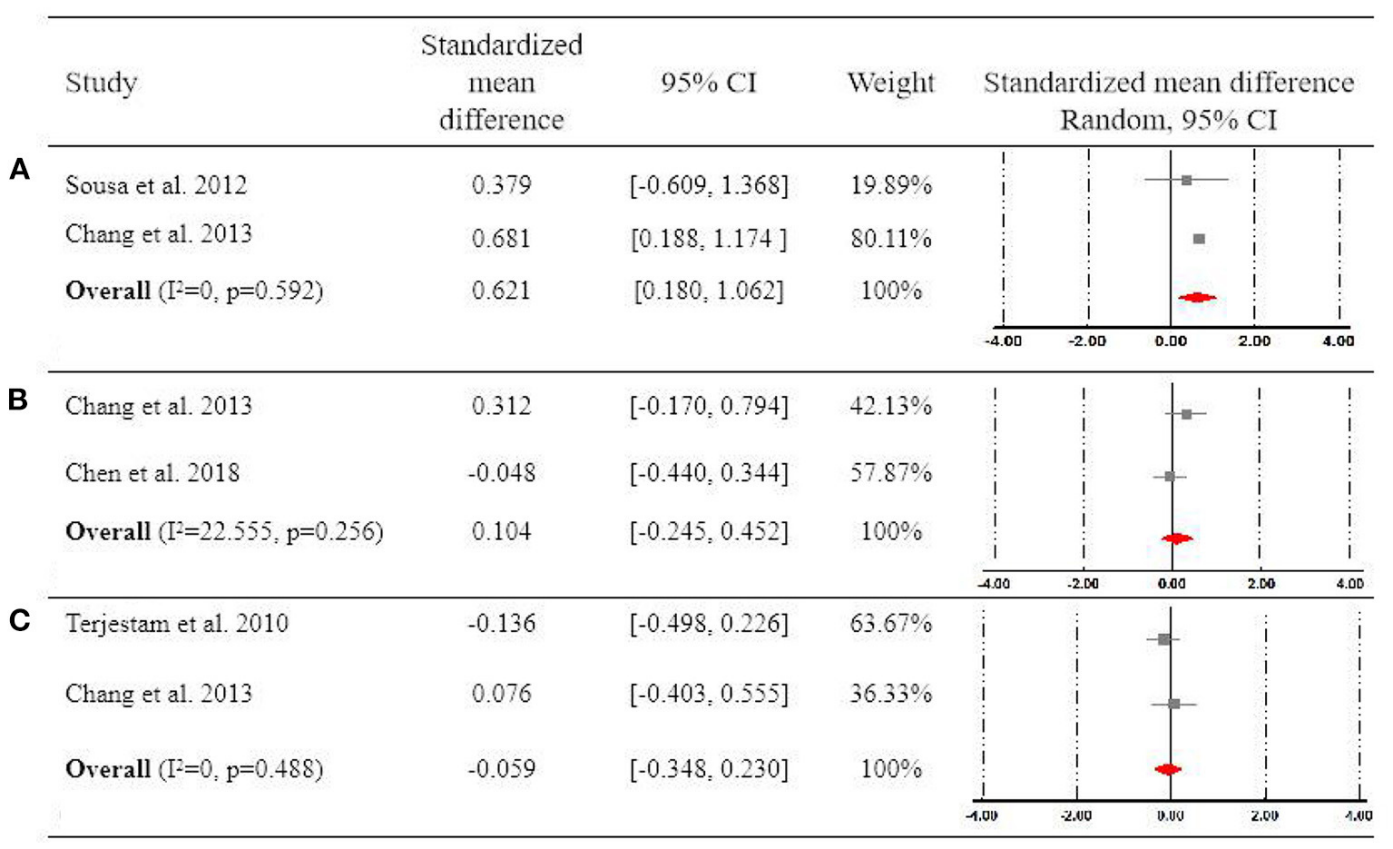
Forest plot for meta-analysis regarding the effect of Qigong on **(A)** saliva cortisol level, **(B)** mood, and **(C)** self-esteem.

Two studies compared the effect of Qigong vs. waitlist condition on changes of cortisol level, which is the biomarker reflecting the stress response (Sousa et al., 2012; Chang et al., 2013). Their pooled results showed that Qigong exercise significantly reduced the cortisol level compared to waitlist controls (SMD = 0.621, 95 CI% [0.180, 1.062]), with a high degree of homogeneity (*I*^2^ = 0%) (Figure 3A).

#### Other Psychological Outcomes

Two studies examined the effect of Qigong vs. waitlist on mood (Chang et al., 2013; Chen and Zheng, 2018). Chen and Zheng (2018) reported the findings on boys and girls, separately, of which the results were combined and analyzed as a unit for meta-analysis. The pooled results of these two studies did not detect the significant difference between Qigong exercise and waitlist (SMD = 0.104, 95 CI% [−0.245, 0.452]), with a low degree of heterogeneity (*I*^2^ = 22.555%) (Figure 3B).

Two studies examined the effect of Qigong exercise vs. waitlist on improving self-esteem (Terjestam et al., 2010; Chang et al., 2013). Their pooled results did not show a significant effect of Qigong on enhancing self-esteem (SMD = −0.059, 95 CI% [−0.348, 0.230]), with a high degree of homogeneity (*I*^2^ = 0%) (Figure 3C).

Some related outcomes were only studied once. For example, Terjestam et al. (2010) suggested that Qigong exercise did not result in significant improvement in psychological distress (SMD = 0.317, 95 CI% [−0.047, 0.681]). Significant enhancement in self-concept was found after 1-year Tai Chi intervention in Bao and Jin (2015), with overall effect size of (SMD = 0.499, 95 CI% [0.165, 0.834]). Additionally, Bao and Niu (2018) focused on the effect of Tai Chi on middle school students' general mental health which included a wide range of dimensions, such as emotional instability and psychological imbalance. Significant improvement in general mental health was also reported with overall effect size of (SMD = −0.377, 95 CI% [−0.641, −0.114]).

## Discussion

This systematic review and meta-analysis summarizes the effect of Tai Chi and Qigong exercise on improving adolescents' psychological health outcomes. Evidence accrued from RCTs and non-randomized controlled trials indicates Tai Chi and Qigong exercise – both short and long-term – appears to have potential mental health benefits in improving psychological symptoms (i.e., depression, anxiety), biomarkers of stress response (i.e., cortisol level), and psychological well-being (i.e., self-concept). The findings in adolescents are partially consistent with previous reviews in various populations (Wang et al., 2010, 2013a, 2014b; Yin and Dishman, 2014; Liu et al., 2015; Sharma and Haider, 2015; Tong et al., 2018).

Anxiety and depression have been extensively focused on in previous epidemiological studies examining the association between exercise and mental health. Congruent with previous systematic reviews of other exercise interventions, such as aerobic exercise (Lees and Hopkins, 2013), yoga (Cramer et al., 2013; Gong et al., 2015; Hendriks et al., 2017), physical activity (Penedoa and Dahna, 2005; Lubans et al., 2016), this review suggested the beneficial effect of Tai Chi and Qigong exercise on reducing anxiety and depression. According to the result of subgroup analysis to compare the difference between Tai chi and Qigong exercise in terms of the effect on psychological symptoms, adolescents practicing Qigong exercise perceived greater improvement in anxiety and depression. It is probably because Qigong focus more on “inside” energy flow while Tai Chi focuses on “outside” defending and attacking intention while practicing (Liu et al., 2015). Specifically, the practice of Qigong focuses on the individual's mind, breath, and inner feeling. Liu et al. (2015) systematically reviewed the effect of Qigong and Tai Chi, respectively, on anxiety and depressive symptoms. Consistent with our results, they found that simply Qigong exercise demonstrated a significant effect on lessening the severity of depressive symptoms. Different findings revealed that Tai Chi and Qigong exercise have the same effect on anxiety and depression symptoms (Jahnke et al., 2010). Wang et al. (2013b) evaluated the effectiveness of Qigong vs. four types of control group on depressive and anxiety symptoms in individuals with anxiety and depression disorders. They found that Qigong was beneficial on depressive symptoms when compared to waiting-list controls or treatment as usual, group newspaper reading, and walking or conventional exercise, and was comparable to that of cognitive-behavioral therapy. However, the beneficial effect of Qigong exercise on anxiety symptoms were not supported by available evidence (Dickerson and Kemeny, 2004). Honestly, the results of this review should be interpreted and generalized cautiously due to the limited number of included studies and the potential heterogeneity across studies. Although the effectiveness of Qigong exercise on anxiety and depression seems to be more prominent, the findings are not conclusive because the studies that were meta-analyzed for anxiety and depression lacked methodological homogeneity in terms variation of Qigong intervention, control group, and outcome measurements. Unfortunately, the comparison of Tai Chi and Qigong exercise to different types of control group was not feasible in this study. Additionally, the meta-analysis was conducted based on various instruments, including both specialized scales measuring anxiety and depression (e.g., State Trait Anxiety Inventory; Nine-item patient health questionnaire depression scale) and integrated scales (e.g., Middle School Students' Mental Health Scale) with subsets of psychological outcomes. These methodological heterogeneities may account for the impact of excluding those studies with high heterogeneity on altering the pooled results and effect sizes on depression symptoms and stress.

Additionally, cortisol has been one of the most frequently studied biomarkers manifesting physiological responses toward social and psychological stress (Dickerson and Kemeny, 2004; Gunnar and Herrera, 2015; Adam et al., 2017). The pooled results of one RCT (Chang et al., 2013) and one NRS (Lee et al., 2009) revealed a favorable effect of Qigong on reducing cortisol level, which reinforces the beneficial effects of Qigong exercise on stress reduction. The findings are in accordance with previous evidence on physical activity in relation to cortisol level among young healthy persons (Alghadir et al., 2015; Hötting et al., 2016). Although the variation between the included studies with regard to experimental design (i.e., RCT vs. NRS) may produce inherent bias of the pooled data, the results suggest that Qigong has the potential to relieve psychological stress by regulating cortisol level. Previous studies mainly focused on identifying dose-response effect of exercise and physical activity for self-perceived psychological outcomes (Tsang et al., 2008; Wang et al., 2009; Roswiyani et al., 2019). Similarly, the included studies rarely used objective measures of psychological outcomes, restricting the possibility to conduct further meta-analysis on these variables. Future studies are encouraged to included objective measures of biomarkers related to stress response and anxiety, such as norepinephrine, epinephrine, blood pressure, and heart rate, to form a more comprehensive understanding of the effect of Qigong on various aspects of psychological health and well-being in adolescents. Little is known regarding the underlying mechanism relating to the beneficial effect of Qigong exercise on psychological health. Possible assumptions claimed that the improvement of psychological status resulted from Qigong practice is probably mediated by the physiological process (e.g., hormone regulation, changes of brain-derived neurotrophic factors) (Litscher et al., 2001). Therefore, measures of relevant biomarkers may help investigate the potential mechanism as well as the reciprocal association between psychological and physiological variables.

Insufficient evidence is provided to confirm the beneficial effect of Qigong on other psychological variables, including mood state and self-esteem. These results may partially contradict with previous systematic review (Wang et al., 2010), which provided reliable evidence supporting the effect of Tai Chi on enhanced mood, but not on self-esteem, among community-dwelling healthy participants and in patients with chronic conditions. Target population may account for the inconsistent findings. Previous RCTs found consistent results that Baduanjin and tai chi exercise did not significantly improve mood and self-esteem, among college students (Li et al., 2015; Zheng et al., 2015). The variation in intervention duration ranging from several weeks to months may explain the inconsistent findings in these variables. For example, considering self-esteem as a relatively stable personal characteristic, it is probably that short-term exposure to intervention is not adequate to produce satisfactory change.

This review appears to support the therapeutic effect of Tai Chi and Qigong exercise on psychological health outcomes in a specified population – adolescents, which has the advantage of acquiring outcomes in particular to this target group compared to various populations involved in previous reviews (Wang et al., 2014b). Due to the limited number of studies, meta-analysis of each indicator only includes two to three trials, resulting in difficulty for data consolidation or synthesis results. The lack of number of studies is aligned with previous meta-analyses in adults in this field (Wang et al., 2013a,b, 2014a). Despite that a growing body of studies examined the psychological effects of Qigong or Tai Chi in recent years, only few of them focused on adolescents specifically. The lack of number of studies and overall unsatisfactory methodological quality prohibit to draw conclusive findings. Additionally, the included studies examined the impact of Tai Chi and Qigong on adolescents' psychological health outcomes, with psychological health outcomes measured primarily in terms of the absence of manifested psychological symptoms and problems, such as depression and anxiety symptoms. This could also be affirmed by the number of trials that are included in meta-analysis, where anxiety and depression were the most frequently measured among all the psychological outcomes. However, relatively few studies have focused on the positive attributes of mental health (Shek, 2007). This is in accordance with less quantitative data for self-esteem and quality of life that can be meta-analyzed. Different from normal adult population, adolescents' psychological well-being has close relation to academic master, peer relationship, and attachment with parents. Future studies with identifying population-based and context-based indicators reflecting the positive aspects of psychological well-being are strongly needed in this population. Additionally, the type of exercise other than Tai Chi or Qigong employed in comparison group was limited. As a result, it is impossible to compare results across studies to recognize whether Qigong exercise, with the emphasis on the movement of the body driven by thoughts and breath, provides equal or superior psychological benefits compared to other types of exercise. Next, the studies included in this review exhibit a variety of Tai Chi or Qigong styles, and frequency, intensity, and duration of intervention. Specifically, the frequency ranges from one to seven sessions per week, and the length of intervention ranges from 7 weeks to 1 year. Few studies examined whether different protocol of Tai Chi or Qigong exercise affected the psychological outcomes. Only one study compared different intensity of Qigong exercise (30 min per session vs. 60 min per session), but they failed to find any significant difference. Further studies with the comparison of different attributes of Tai Chi and Qigong exercise are encouraged to identify the optimized intervention protocol that maximize the dose-response effects and the adherence rate among participants. Finally, moderate to high heterogeneity was found for the overall effects of Qigong and Tai Chi on anxiety, depression, and stress. High variation in instruments to measure outcomes and difference in methodological rigor between RCTs and NRS of included studies may account for the potential sources of heterogeneity. Additionally, publication bias may also contribute to the methodological concern of the included studies as most of them were mainly conducted in China. Well-controlled randomized trials with rigorous study design, larger sample size, and the availability of other types of exercise (e.g., aerobic exercise) as comparison group are called on for future studies to provide complementary evidence in this field.

## Conclusion

The findings of this systematic review only provide preliminary evidence that Qigong exercise may be potentially beneficial for adolescents' psychological well-being, including reduced anxiety and depressive symptoms, and decreased cortisol level. However, the outcomes should be interpreted cautiously due to limited number of studies and methodological weakness. Future studies that are rigorous, prospective, well-controlled randomized trials with appropriate comparison groups, and with validated outcome measure are now needed to draw conclusive findings to understand the effects of Tai Chi and Qigong exercise on psychological well-being. Furthermore, objective measure of anxiety and stress response as well as the consideration of more positive aspects of psychological health outcomes in particular to this population may provide more reliable evidence and inspiration in this field.

## Data Availability Statement

The original contributions presented in the study are included in the article/Supplementary Material, further inquiries can be directed to the corresponding author/s.

## Author Contributions

XL: conceptualization, formal analysis, and writing—original draft. RL: formal analysis and writing—review and editing. JC and FL: screening and formal analysis. LS, XC, and DZ: writing—review and editing. All authors contributed to the article and approved the submitted version.

## Funding

This work was partially supported by a grant from the National Social Science Fund of China [Grant No. 13&ZD140].

## Conflict of Interest

The authors declare that the research was conducted in the absence of any commercial or financial relationships that could be construed as a potential conflict of interest.

## Publisher's Note

All claims expressed in this article are solely those of the authors and do not necessarily represent those of their affiliated organizations, or those of the publisher, the editors and the reviewers. Any product that may be evaluated in this article, or claim that may be made by its manufacturer, is not guaranteed or endorsed by the publisher.
