# Supplementary material for: The Effects of Tai Chi and Qigong Exercise on Psychological Status in Adolescents: A Systematic Review and Meta-Analysis
Source: Front Psychol. 2021 Nov 24;12:746975. doi: 10.3389/fpsyg.2021.746975 (PMC8652254; doi:10.3389/fpsyg.2021.746975)
Supplement: Supplementary file 1 [file Data_Sheet_1.PDF]

Supplementary materials

List of content:

- Additional file 1. Search terms used in each database

- Additional file 2. Checklist to Evaluate a Report of a Nonpharmacological Trial (CLEAR-NPT)

Additional file 1.

Table 1. Search strategy in Medline via Pubmed.

Search	Query	Items found
#1	Search ((((((((((((((((((((((Qigong[MeSH Terms]) OR Tai Ji[MeSH Terms]) OR Qigong[Title/Abstract]) OR Tai Ji[Title/Abstract]) OR Ch'i Kung[Title/Abstract]) OR Qi-gong[Title/Abstract]) OR Chi Kung[Title/Abstract]) OR Chi Chung[Title/Abstract]) OR Qi Chung[Title/Abstract]) OR Qi-training[Title/Abstract]) OR Chi Gong[Title/Abstract]) OR Qigong Massage[Title/Abstract]) OR Tai-ji[Title/Abstract]) OR Tai Chi[Title/Abstract]) OR Tai Ji Quan[Title/Abstract]) OR Taiji[Title/Abstract]) OR Taijiquan[Title/Abstract]) OR T'ai Chi[Title/Abstract]) OR Tai Chi Chuan[Title/Abstract]) OR Tai Chi Chih[Title/Abstract]) OR Tai Chi Qigong[Title/Abstract]) OR Baduanjin[Title/Abstract]) OR Qi Gong[Title/Abstract]	2631
#2	Search (((((((Depression[MeSH Terms]) OR Anxiety[MeSH Terms]) OR Depression[Title/Abstract]) OR Anxiety[Title/Abstract]) OR Psychological well-being[Title/Abstract]) OR Mental[Title/Abstract]) OR Stress[Title/Abstract]) OR Mood[Title/Abstract]	1485556
#3	Search (((((((((Adolescent[MeSH Terms]) OR Youth[Title/Abstract]) OR Student*[Title/Abstract]) OR Teenager*[Title/Abstract]) OR Child[MeSH Terms]) OR Children[Title/Abstract]) OR Childhood[Title/Abstract]) OR Adolescent[Title/Abstract]) OR Child[Title/Abstract]) OR Students[MeSH Terms]	3619205
#4	#1 and #2 and #3	72

Table 2. Search strategy in Embase and PsychInfo via Ovid.

Search	Query	Items found
1	exp Qigong/	699
2	exp Tai Ji/	2817
3	Qigong.ab,kw,ti.	1262
4	Tai Ji.ab,kw,ti.	99
5	Qi Gong.ab,kw,ti.	243
6	Ch'i Kung.ab,kw,ti.	26
7	Qi-gong.ab,kw,ti.	243
8	Chi Kung.ab,kw,ti.	26
9	Chi Chung.ab,kw,ti.	9
10	Qi Chung.ab,kw,ti.	0
11	Qi-training.ab,kw,ti.	167
12	Chi Gong.ab,kw,ti.	13
13	Qigong Massage.ab,kw,ti.	19
14	Tai Chi.ab,kw,ti.	2936
15	Tai Ji Quan.ab,kw,ti.	48
16	Taiji.ab,kw,ti.	133
17	Taijiquan.ab,kw,ti.	66
18	T'ai Chi.ab,kw,ti.	2936
19	Tai Chi Chuan.ab,kw,ti.	304
20	Tai Chi Chih.ab,kw,ti.	45
21	Tai Chi Qigong.ab,kw,ti.	87
22	Baduanjin.ab,kw,ti.	101
23	Tai-ji.ab,kw,ti.	99
24	1 or 2 or 3 or 4 or 5 or 6 or 7 or 8 or 9 or 10 or 11 or 12 or 13 or 14 or 15 or 16 or 17 or 18 or 19 or 20 or 21 or 22 or 23	5223

25	exp Depression/	490318
26	exp Anxiety/	274323
27	Depression.ab,kw,ti.	702218
28	Anxiety.ab,kw,ti.	459254
29	Mental.ab,kw,ti.	814892
30	Psychological well-being.ab,kw,ti.	23733
31	Mental.ab,kw,ti.	814892
32	Stress.ab,kw,ti.	1156661
33	Mood.ab,kw,ti.	179128
34	25 or 26 or 27 or 28 or 29 or 30 or 31 or 32 or 33 or 34	2881777
35	exp Adolescent/	1498276
36	exp Child/	2573139
37	exp Students/	508560
38	Adolescent.ab,kw,ti.	270653
39	Youth.ab,kw,ti.	167935
40	"Student*".ab,kw,ti.	856928
41	"Teenager*".ab,kw,ti.	28517
42	Children.ab,kw,ti.	1774875
43	Childhood.ab,kw,ti.	423154
44	Child.ab,kw,ti.	697821
45	35 or 36 or 37 or 38 or 39 or 40 or 41 or 42 or 43 or 44	5284545
46	24 and 34 and 45	141

Table 3. Search strategy in CINAHL, ERIC, and SPORTDiscus via EBSCOhost.

#	Query	Results
S1	(MH “Qigong”)	472
S2	(MH “Tai Chi”)	1,502
S3	TI “Qigong” OR AB “Qigong” OR TI “Tai Ji” OR AB “Tai Ji” OR TI “Tai Chi” OR AB “Tai Chi” OR TI “Ch'i Kung” OR AB “Ch'i Kung” OR TI “Qi-gong” OR AB “Qi-gong” OR TI “Chi Kung” OR AB “Chi Kung”	3,897
S4	TI “Qi Gong” OR AB “Qi Gong” OR TI “Chi Chung” OR AB “Chi Chung” OR TI “Qi Chung” OR AB “Qi Chung” OR TI “Qi-training” OR AB “Qi-training” OR TI “Chi Gong” OR AB “Chi Gong” OR TI “Qigong Massage” OR AB “Qigong Massage”	283
S5	TI “Tai-ji” OR AB “Tai-ji” OR TI “Tai Ji Quan” OR AB “Tai Ji Quan” OR TI “Taiji” OR AB “Taiji” OR TI “Taijiquan” OR AB “Taijiquan” OR TI “T'ai Chi” OR AB “T'ai Chi” OR TI “Tai Chi Chuan” OR AB “Tai Chi Chuan”	1,179
S6	TI “Tai Chi Chih” OR AB “Tai Chi Chih” OR TI “Tai Chi Qigong” OR AB “Tai Chi Qigong” OR TI “Baduanjin” OR AB “Baduanjin”	263
S7	S1 OR S2 OR S3 OR S4 OR S5 OR S6	5,011
S8	(MH “Depression”)	68,577
S9	(MH “Anxiety”)	20,662
S10	TI “Depression” OR AB “Depression” OR TI “Anxiety” OR AB “Anxiety” OR TI “Psychological well-being” OR AB “Psychological well-being” OR TI “Mental” OR AB “Mental” OR TI “Stress” OR AB “Stress” OR TI “Mood” OR AB “Mood”	627,233
S11	S8 OR S9 OR S10	650,068
S12	TI “Adolescent” OR AB “Adolescent” OR TI “Youth” OR AB “Youth” OR TI “Student*” OR AB “Student*” OR TI “Teenager*” OR AB “Teenager*” OR TI “Child” OR AB “Child” OR TI “Children” OR AB “Children”	1,823,949
S13	TI “Childhood” OR AB “Childhood”	123,203

S14	S12 OR S13	1,866,469
S15	S7 AND S11 AND S14	84

Table 4. Search strategy in the Cochrane Central Register of Controlled Trials (CENTRAL)

Search	Query	Items found
#1	MeSH descriptor: [Qigong] explode all trees	65
#2	(Qigong):ti,ab,kw (Word variations have been searched)	442
#3	(Ch'i Kung):ti,ab,kw (Word variations have been searched)	0
#4	(Qi-gong):ti,ab,kw (Word variations have been searched)	53
#5	(Chi Kung):ti,ab,kw (Word variations have been searched)	11
#6	(Qi Gong):ti,ab,kw (Word variations have been searched)	57
#7	(Chi Chung):ti,ab,kw (Word variations have been searched)	6
#8	(Qi Chung):ti,ab,kw (Word variations have been searched)	1
#9	(Qi-training):ti,ab,kw (Word variations have been searched)	11
#10	(Chi Gong):ti,ab,kw (Word variations have been searched)	18
#11	(Qigong Massage):ti,ab,kw (Word variations have been searched)	13
#12	MeSH descriptor: [Tai Ji] explode all trees	342
#13	(Tai Ji):ti,ab,kw (Word variations have been searched)	367
#14	(Tai-ji):ti,ab,kw (Word variations have been searched)	359
#15	(Tai Chi):ti,ab,kw (Word variations have been searched)	1134
#16	(Tai Ji Quan):ti,ab,kw (Word variations have been searched)	24
#17	(Taiji):ti,ab,kw (Word variations have been searched)	36
#18	(Taijiquan):ti,ab,kw (Word variations have been searched)	85
#19	(T'ai Chi):ti,ab,kw (Word variations have been searched)	38
#20	(Tai Chi Chuan):ti,ab,kw (Word variations have been searched)	107
#21	(Tai Chi Chih):ti,ab,kw (Word variations have been searched)	36
#22	(Tai Chi Qigong):ti,ab,kw (Word variations have been searched)	74
#23	(Baduanjin):ti,ab,kw (Word variations have been searched)	101

#24	#1 OR #2 OR #3 OR #4 OR #5 OR #6 OR #7 OR #8 OR #9 OR #10 OR #11 OR #12 OR #13 OR #14 OR #15 OR #16 OR #17 OR #18 OR #19 OR #20 OR #21 OR #22 OR #23	1724
#25	MeSH descriptor: [Depression] explode all trees	11708
#26	(Depression):ti,ab,kw (Word variations have been searched)	80937
#27	MeSH descriptor: [Anxiety] explode all trees	7573
#28	(Anxiety):ti,ab,kw (Word variations have been searched)	47522
#29	(Psychological well-being):ti,ab,kw (Word variations have been searched)	5737
#30	(Mental):ti,ab,kw (Word variations have been searched)	54262
#31	(Stress):ti,ab,kw (Word variations have been searched)	55795
#32	(Mood):ti,ab,kw (Word variations have been searched)	19819
#33	#25 OR #26 OR #27 OR #28 OR #29 OR #30 OR #31 OR #32	187053
#34	MeSH descriptor: [Adolescent] explode all trees	101062
#35	(Adolescent):ti,ab,kw (Word variations have been searched)	130043
#36	(Youth):ti,ab,kw (Word variations have been searched)	6779
#37	MeSH descriptor: [Students] explode all trees	4103
#38	(Student*):ti,ab,kw (Word variations have been searched)	33179
#39	(Teenager*):ti,ab,kw (Word variations have been searched)	883
#40	MeSH descriptor: [Child] explode all trees	2623
#41	(Child):ti,ab,kw (Word variations have been searched)	137479
#42	(Children):ti,ab,kw (Word variations have been searched)	137478
#43	(Childhood):ti,ab,kw (Word variations have been searched)	15119
#44	#34 OR #35 OR #36 OR #37 OR #38 OR #39 OR #40 OR #41 OR #42 OR #43	250346
#45	#24 AND #33 AND # 44	60

Additional file 2. Checklist to Evaluate a Report of a Nonpharmacological Trial (CLEAR-NPT)

	**Criterion**	**Yes**	**No**	**Unclear**	**Comment/Note**
1	Was the generation of allocation sequences adequate?				The investigator should describe a random component in sequence generation process.
2	Was the treatment allocation concealed?				Adequate concealment of allocations prior to assignment
3	Were details of the intervention administered to each group stated or made available?				Did the investigator describe the intervention administered to each group clearly?
4	Were care providers experiences or skills in each arm (group) appropriate?				Did the investigator describe the experiences or skills of care providers in each group?
5	Was participant adherence or compliance assessed quantitatively?				Did the investigator quantitatively measured how the participants followed the intervention protocol?
6	Were participants adequately blinded?<br>❖ If NO, please go to 7.1. and 7.2.				The answer is usually NO in nonpharmacological studies
6.1	Were other treatments and care (i.e., co-interventions) the same in each randomized group?				If there exited co-interventions, did each group get same amount of time/attention of co-interventions?
6.2	Were withdrawals and lost-to-follow-up the same in each randomized group?				Calculate the difference of drop-out to see if it is statistically significant.

7	Were care providers for the participants adequately blinded?<br>❖ If NO, please go to 8.1. and 8.2.				The answer is usually NO in nonpharmacological studies
7.1	Were all other treatments and care (co-interventions) the same in each randomized group?				Were the care providers of each group had equivalent experiences of training?
7.2	Were withdrawals and lost-to-follow up the same in each randomized group?				Calculate the difference of drop-out to see if it is statistically significant.
8	Were outcome assessors adequately blinded to assess the primary outcomes?				If participants completed self-reported study forms, this item becomes N/A.
8.1	If outcome assessors were not adequately blinded, were specific methods used to avoid ascertainment bias (systematic differences in outcome assessment)?				Did the investigator describe possible and study-specific methods to avoid assessor bias?
9	Was the follow-up schedule the same in each group? (parallel design)				Did participants in each group follow the same timeline or were there variations?
10	Were the main outcomes analyzed according to the intention-to-treat principle?				Intention-to-treat analysis should include everyone who was randomized into the study, no matter if they completed the study or not.
